# Effects of positive mood on attentional breadth for emotional stimuli

**DOI:** 10.3389/fpsyg.2014.01277

**Published:** 2014-11-11

**Authors:** Maud Grol, Rudi De Raedt

**Affiliations:** Department of Experimental Clinical and Health Psychology, Ghent University, Ghent, Belgium

**Keywords:** positive emotions, emotional information, attentional breadth, depressive symptoms, individual differences

## Abstract

Although earlier studies have related positive emotions to attentional broadening, recent findings point out the complexity of this relation and show that these broadening effects interact with factors such as characteristics of the information that is presented. Besides stimuli characteristics, individual characteristics such as the presence of depressive symptoms could also influence the broadening effects as depressive symptoms have previously been related to a more narrow attentional scope. Therefore, the aim of this study was to further investigate the attentional broadening effects of positive emotions, testing whether this is influenced by the emotional valence of the information presented and secondly, how the presence of depressive symptoms might interact with this relationship. We used a performance-based measure to assess fluctuations in attentional broadening for positive, neutral, and negative stimuli. We assessed the presence and severity of depressive symptoms in an unselected study sample and tested whether these symptoms moderate the relationship between induced positive mood and attentional breadth for emotional information. Results showed no direct relation between positive mood and attentional breadth, regardless of the emotional valence of the stimuli. However, the presence of depressive symptoms moderated this relationship in such a way that among low levels of depressive symptoms, positive mood was related to attentional broadening specifically when positive information was presented, while at high levels of depressive symptoms this relation was reversed. The current findings suggest that both stimuli characteristics, individual characteristics and their interplay should be taken into account when investigating the broadening effects of positive emotions.

## INTRODUCTION

The broaden-and-build theory (Fredrickson, 1998, 2001) states that negative and positive emotions have distinct cognitive and psychophysiological effects. Whereas negative emotions are associated with narrowed thought–action repertoires and action tendencies, positive emotions would broaden one’s thought–action repertoire which over time contributes to building personal resources. Studies investigating these broadening effects of positive emotions have found evidence showing that positive emotional experiences are related to more flexible cognitive categorization, processing of material in a more integrated manner (e.g., Isen and Daubman, 1984), increased creativity, and social openness (Isen and Daubman, 1984; Isen et al., 1987; Baas et al., 2008; Garland et al., 2010). Although these studies involve a wide range of conceptualizations of broadened thought–action repertoires, mostly targeting higher-level cognition, several studies have also investigated the effects of positive emotions on visual attentional breadth. Visual attentional breadth, or the attentional scope, has mostly been measured using global–local visual processing and attention selection tasks. Global–local processing tasks measure the extent to which individuals attend to global features (the whole) and local features (parts) of a composite stimulus (Navon, 1977; Kimchi and Palmer, 1982). Effects on attention selection have been assessed with an adjusted version of the Eriksen flanker task (Eriksen and Eriksen, 1974; Rowe et al., 2007) which allows investigation of interference of non-target stimuli on the response to the central target at close to further distances. Several studies have shown that positive emotions are associated with a more broadened attentional scope (Derryberry and Tucker, 1994; Gasper and Clore, 2002; Gasper, 2004; Fredrickson and Branigan, 2005; Rowe et al., 2007). Furthermore, optimism has also been associated with more broadened attention (Basso et al., 1996). On the other hand, negative mood and depressive symptoms have been associated with a more narrowed attentional scope (Derryberry and Tucker, 1994; Basso et al., 1996; De Fockert and Cooper, 2014).

Interestingly, the broaden-and-build theory (Fredrickson, 1998, 2001) proposes the effects of positive emotions on attentional broadening to be an underlying mechanism in the relationship between positive emotions and resilience. Because attention can be considered as the start of information processing, it is likely to influence further information processing (e.g., memory and interpretation). More narrowed attention, as a result of (chronic) negative mood, may allow more elaborate and deep encoding of information at the center of attention; however, it will also make it more difficult to disengage from or forget this information. This is consistent with studies associating depression with increased difficulties in disengaging attention from (mood-congruent) negative information and increased accessibility of negative material in memory (for reviews, see De Raedt and Koster, 2010; Gotlib and Joormann, 2010). So while threatening situations that require quick and decisive action might benefit from more narrowed attention (Fredrickson, 1998) as it will reduce distractibility from irrelevant information (Friedman and Förster, 2010), this may become dysfunctional when situational demands (or opportunities) change. Positive emotions on the other hand, broaden attention and allow more flexible processing of a wider range of information (Fredrickson, 1998, 2001, 2004; Whitmer and Gotlib, 2013). This more broadened attentional processing style could (over time) counteract the cognitive impairments related to depression and protect against a depressive responding to stress (Fredrickson, 1998, 2001).

The effects of positive emotions on attentional broadening might thus serve an adaptive function. However, results on attentional broadening have been mixed (Bruyneel et al., 2013) and may be conditional to other factors or processes. It has been suggested that the effects of emotion on attentional breadth depend on motivational factors related to mood or emotional information (Gable and Harmon-Jones, 2008, 2010). Positive mood and processing of information low in approach motivation were shown to be related to attentional broadening, whereas positive mood and information (appetitive stimuli) high in approach motivation were related to a decrease in attentional broadening (Gable and Harmon-Jones, 2008). Furthermore, it has also been shown that the attentional broadening effects of positive mood interact with characteristics of the presented stimuli in the target of attention itself, such as the self-relevance of the stimulus (Grol et al., 2014) or the emotional valence (Wadlinger and Isaacowitz, 2006). Wadlinger and Isaacowitz (2006) applied eye-tracking while three similarly valenced images (either three high, medium, or low positive, three neutral, or three high, medium, or low negative images) were presented in varying central–peripheral arrays. They operationalized attentional broadening as more fixations on peripheral images. Results showed positive mood induction to be related to more fixations on peripheral images, but only when high positive stimuli were presented (Wadlinger and Isaacowitz, 2006). In line with the proposed effect of positive emotions on attentional broadening (Fredrickson, 1998, 2001), it may thus be that emotional stimuli in the target of attention also influence attentional breadth itself (see also Grol and De Raedt, 2014), and that the emotional valence of the presented information interacts with the induced mood state. Measuring attentional breadth when positive or negative information is in the target of attention could influence the effect of positive mood on attentional broadening.

The above described research findings considering factors of influence on attentional broadening by positive emotions, underscore the apparent complexity of the effects and point out the need for further research. Therefore, in this study we further investigated the relationship between positive mood and attentional broadening and how this interacts with the emotional valence of the presented information that is in the target of attention. Based on earlier findings (Wadlinger and Isaacowitz, 2006) we expected that positive mood would be related to attentional broadening for positive information but that the effect of positive mood on attentional broadening could be hampered when neutral or negative information is presented.

Besides studying how characteristics of the presented stimuli influence the attentional broadening effects of positive emotions, it is also relevant to investigate how personal characteristics may influence these broadening effects. The broaden-and-build theory proposes that the broadening effects play an underlying role in the relation between positive emotions and resilience (Fredrickson, 1998, 2001), which suggests that especially resilient people can benefit from positive emotions in the form of attentional broadening. In turn, this may also suggest that people who are vulnerable to develop mood disorders, for example, reflected in the presence of depressive symptoms (Fergusson et al., 2005), may be less able to benefit from positive emotions in the form of attentional broadening. Moreover, previous studies have shown that depressive symptoms are related to a more narrowed attentional scope (Basso et al., 1996; De Fockert and Cooper, 2014). Thus it is possible that the presence of depressive symptoms could hamper the effects of positive emotions on attentional broadening. Furthermore, it should be considered that in the context of mood disorders certain types of information are specifically relevant. In mood disorders such as depression, attentional processes are often affected depending on the processing of specific types of information. There is a great wealth of research associating depression with heightened self-focused attention (Mor and Winquist, 2002) and increased difficulties in disengaging attention from negative information once it captured attention (for reviews, see De Raedt and Koster, 2010; Gotlib and Joormann, 2010). Although the processing of this kind of (meaningful) information could also affect attentional processes in healthy individuals, this may further interact with the presence of depressive symptoms given the attentional bias in depression for such information. Therefore, we measured the presence of depressive symptoms and examined if this would moderate the relation between positive mood and visual attentional breadth for emotional material.

In this study, we examined the effects of positive mood on attention for emotional information with a performance-based measure of visuospatial attentional breadth. Wadlinger and Isaacowitz (2006) operationalized attentional broadening for emotional information as more fixations on peripheral images. However, it is possible that participants still displayed a narrowed attentional focus but on more peripheral images on the screen, rather than a broadening of the visuospatial attentional scope where more stimuli (in center and periphery) fall within the attentional field. Our performance-based measure of visuospatial attentional breadth has been successfully used before to measure fluctuations in attentional broadening/narrowing related to centrally presented, personally relevant information (Bosmans et al., 2009) and self vs not-self-related information (Grol et al., 2014). The measure has a dual-task nature in the sense that participants both have to identify a central emotional stimulus and localize a peripheral target which are presented simultaneously. Central and peripheral stimuli are both task relevant, so one cannot ignore peripheral information or central information to perform the task. To induce positive mood, we used a mood induction procedure (MIP) based on imagining autobiographical memories of events, which is less likely to induce high approach motivated positive mood as compared to using appetitive stimuli or approach motivating cues (Gable and Harmon-Jones, 2008).

We measured visuospatial attentional breadth for positive, neutral, and negative information using happy, neutral, and sad facial stimuli. Based on previous research linking positive mood to attentional broadening, but considering the results of the study by Wadlinger and Isaacowitz (2006), we hypothesized positive mood induction, as compared to neutral mood induction, to be related to a broadening of visuospatial attention only when positive information was presented. Furthermore, given the relation between depressive symptoms and a more narrowed attentional scope (Basso et al., 1996; De Fockert and Cooper, 2014) we also investigated whether depressive symptoms moderated the effects of positive mood on attentional breadth for emotional material, hypothesizing that higher levels of depressive symptoms hamper the relation between positive mood induction and attentional broadening.

## MATERIALS AND METHODS

### PARTICIPANTS

Forty undergraduate students (36 females) aged between 18 and 26 years (M = 20.80, SD = 1.87) participated in this experiment in exchange for a financial reward (€8). This experiment was approved by the local ethical committee of the Faculty of Psychology at Ghent University. Participants provided written informed consent via a consent form which was approved by the ethical committee of the Faculty of Psychology at Ghent University.

### MATERIALS

#### Questionnaire measures^1^

Mood before and after the mood induction was measured with the Positive and Negative Affect Schedule (PANAS; Watson et al., 1988) measuring how participants were feeling “*at this moment*.” Participants were asked to give their ratings on a 5-point Likert scale ranging from 1 “very slightly” to 5 “very much.”

The presence and severity of depressive symptoms was measured using the self-report Beck Depression Inventory (BDI-II; Beck et al., 1996; Van der Does, 2002). This self-report scale consists of statements (responses ranging from 0 to 3) and participants are asked to pick out the response that best fits the way the participant has been feeling during the past 2 weeks.

The tendency to use mental imagery in daily life was assessed using the 12 item Spontaneous Use of Imagery Scale (SUIS; Reisberg et al., 2003; Nelis et al., 2014). Participants rated the degree to which descriptions are appropriate for them on a 5-point scale ranging from 1 “never appropriate” to 5 “always completely appropriate.”

#### Mood induction procedure

The MIP consisted of a procedure in which mental imagery is used. Participants were instructed to vividly imagine either a self-provided neutral- or happy-inducing autobiographical memory. All participants first practiced the use of mental imagery from a field perspective (i.e., first person perspective) by completing an imagery practice task of cutting a lemon (Holmes et al., 2008). Following the practice task, participants in the neutral condition were instructed to recall a memory of a specific event that did not elicit strong negative or positive emotions at that time, while participants in the positive condition were instructed to recall a memory of a specific event that happened more than 1 week ago which made them feel very happy at that time. Participants were asked to shut their eyes while describing what they remembered in detail. Participants received instructions (Watkins and Moberly, 2009; based on Holmes et al., 2008) to promote concreteness and imagining the event from a field perspective. They imagined the event for 30 s after which they were asked a series of questions (based on Watkins and Moberly, 2009), asking them to focus on what they could see, hear, and physically feel. Following these questions, participants were instructed to continue imagining the event for another 30 s without describing aloud. During the mood induction, manipulation checks were administered to measure the use of field and observer perspective. Participants were asked to rate on a 5-point Likert scale the extent to which they adopted a field perspective, that is the extent to which they “saw the event through their own eyes and were actively involved?” and the extent to which they adopted an observer perspective, “to what extent did you experience the event looking at yourself from outside, as if you see yourself taking part in the situation?” In order to make the induction of the desired mood more lasting throughout the experimental task, music was played during imagining the autobiographical memory and continued playing throughout the task. In the positive condition we used Mike Oldfield’s “Music of the spheres” (track 2, 3, 5, and 6). In the neutral condition we used Chopin’s “Waltzes Nos. 11 and 12,” which have been successfully used to induce neutral mood in previous studies (Startup and Davey, 2001; Heene et al., 2007).

#### Experimental task

**FIGURE 1:**
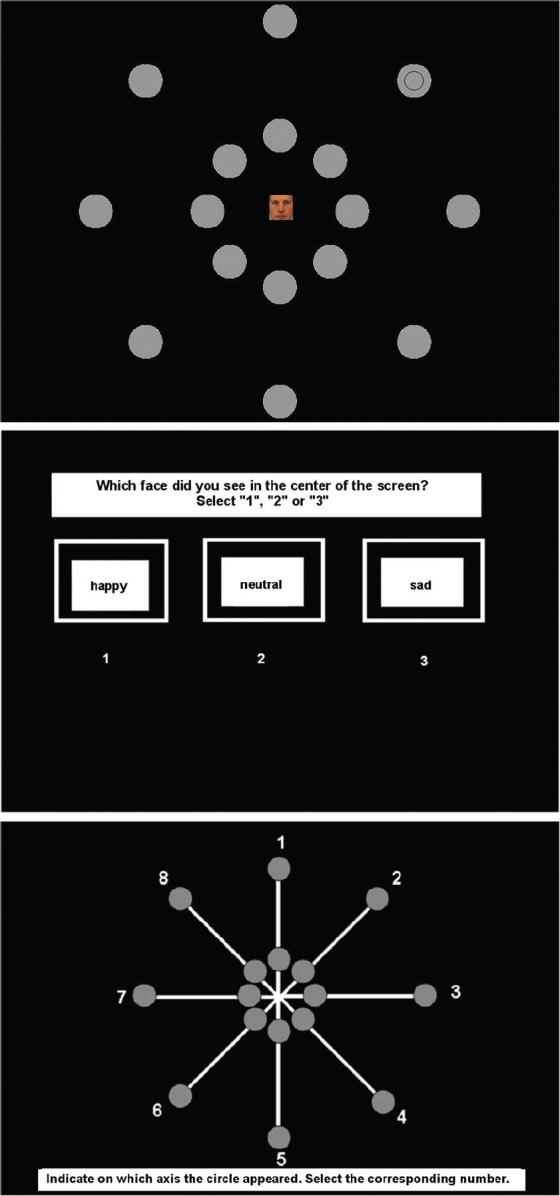
**Stimulus presentation of the Attentional Breadth Task.** The gray dots are presented in pairs of two, simultaneously with the central face and the target stimulus. The first response screen asks participants which face they have seen. The second response screen asks participants on which of eight axes the target stimulus was presented.

Attentional breadth for emotionally valenced stimuli was measured using an adaptation of a task measuring attentional breadth in relation to centrally presented stimuli (Bosmans et al., 2009). Participants were seated at a distance of 27 cm from a 19” CRT-computer screen, using a chin rest to ensure correct positioning. In each trial, a picture of an emotional face without hairline (82 × 82 pixels) appeared in the center of the screen (see Figure 1). Faces were taken from the Karolinska Directed Emotional Faces database (Lundqvist et al., 1998). For each condition (i.e., positive, neutral, negative) we selected eight faces. The selection was based on a valence and arousal rating (low arousal = 1; high arousal = 9) obtained from prior validation (Goeleven et al., 2008) and an equal number of male and female faces was selected within each condition. The selected faces were positive (happy; mean arousal = 3.73, SD = 0.29), neutral (neutral; mean arousal = 2.37, SD = 0.15), and negative (sad; mean arousal = 3.51, SD = 0.33). The positive and negative faces did not differ in level of arousal (t < 1.5). Simultaneously with presentation of the central face, 16 gray dots with a diameter of 2 cm appeared around the face in two concentric circles (see Figure 1). One circle appeared at 4.5 cm from the central face at 10^°^ of the visual angle, the other circle appeared at 11.2 cm from the central face at 25^°^ of the visual angle. The gray dots were arranged in pairs of two, one close and one far, situated on one of eight imperceptible axes. Simultaneously with presentation of the face and gray dots, a smaller black circle with a diameter of 1.3 cm appeared in one of the gray dots, either close or far. All stimuli were presented for 68 ms in order to prevent confounds of saccadic eye movements in search of the peripheral target (Ball et al., 1988).

Participants were instructed to both correctly identify the expressed emotion of the central face (i.e., happy, neutral, sad) and correctly localize the black circle. After the simultaneous presentation of the stimuli, participants were asked to identify the central stimulus (see Figure 1). Participants had no time limit to give their response. After their response, they were asked to identify the axis on which the target stimulus (i.e., the smaller black circle) had appeared (see Figure 1). Again, participants had no time limit to give their response. The accuracy on localizing this peripheral target was used as the main dependent variable. The proportion of correctly localized target stimuli was calculated based on trials in which participants also correctly identified the central stimulus to make sure participants maintained attention to the center of the screen during the task.

The task consisted of eight practice trials with a presentation time of 250 ms, followed by eight practice trials with a presentation time of 68 ms similar to the test phase. The test phase consisted of 144 trials, with six types of trials: Positive close, Positive far, Neutral close, Neutral far, Negative close, and Negative far which were randomly presented in three blocks consisting of 48 trials each.

### PROCEDURE

Participants were randomized to receive either the positive or the neutral MIP. After written informed consent, baseline levels of mood were measured. Following this, participants completed the MIP procedure and mood was measured again immediately afterward. After the MIP, participants performed the experimental task. Finally, participants were administered the additional questionnaires.

### DATA ANALYSIS

To assess the effect of the MIP on mood, a mixed ANOVA with Group (Positive MIP vs Neutral MIP) as between subject factor and Time (before vs after the MIP) as within-subject factor was performed on the PANAS positive scale and negative scale separately.

For the analyses of the attentional breadth task, all trials were deleted in which the central face was incorrectly identified, to make sure participants also maintained attention to the center of the screen during the task. We could then calculate an Attentional Narrowing Index (ANI = accuracy target stimulus close to face - accuracy target stimulus far from face) for positive trials (ANIpositive), neutral trials (ANIneutral), and negative trials (ANInegative) separately. Although our current research question focuses on attentional *broadening* we calculated an ANI as for the original task in Bosmans et al. (2009) they calculated the ANI. To keep the outcome index consistent across studies (Bosmans et al., 2009; Grol and De Raedt, 2014; Grol et al., 2014), in order to compare across studies, we also calculated the ANI.

In total an average of 5.47% of the trials was deleted due to incorrect identification of the central face. Participants were excluded from further analysis if the number of deleted trials for any of the different trial types (Positive close, Positive far, Neutral close, Neutral far, Negative close, and Negative far) was more than 50%. This resulted in excluding two participants from further analysis, one participant from the positive MIP condition and one from the neutral MIP condition. As data on the percentage of deleted trials was not normally distributed, we performed a non-parametric Mann–Whitney test to check for group differences in the number of deleted trials per trial type. This test showed no differences between groups in terms of the percentages of trials that were deleted for any of the trial types, all p-values >0.10. Across all participants there were differences in accuracy for identifying the central stimulus depending on the emotional valence of the presented stimulus, non-parametric Friedman’s ANOVA yielded a significant effect, χ^2^ (2, *N* = 38) = 22.61, *p* < 0.001. Follow-up Wilcoxon’s signed-rank tests show that participants made less errors when identifying the positive faces compared to neutral faces, *T* = 51.50, *p* < 0.001, *r* = -0.40, or negative faces, *T* = 35, *p* < 0.001, *r* = -0.49. Participants also made less errors when identifying neutral faces compared to negative faces, *T* = 131, *p* = 0.006, *r* = -0.31. However, non-parametric Spearman’s rho correlations showed that the accuracy rates on identifying the central stimuli were not related to the ANI for neither positive stimuli, neutral stimuli, nor negative stimuli, all p-values >0.20.

To look at the effect of the positive mood manipulation on attentional breadth for valenced stimuli we performed a 3 Valence (Positive vs Neutral vs Negative) × 2 Distance (far vs close) × Group (Positive MIP vs Neutral MIP) mixed ANOVA with the proportion of correctly localized peripheral targets as the dependent variable. We also investigated the direct effect of positive mood on attentional breadth by including the change in positive mood across the induction (i.e., the difference score across all participants) as a predictor into the analysis, which enhances power considerably. We performed a 3 Valence (Positive vs Neutral vs Negative) × 2 Distance (far vs close) mixed ANOVA on the proportion of correctly localized peripheral targets, with the change in positive mood across the mood induction (ΔPANASpositive) as a continuous independent variable (i.e., covariate). This latter analysis using a continuous independent variable allows us to take into account interindividual differences in the effectiveness of the MIP and to more directly test the influence of positive mood changes.

We aimed to test the moderating effect of the presence of depressive symptoms, as measured with the BDI, on the relation between positive mood and attentional breadth and whether this further interacted with the Valence of the central stimuli. To test such moderation effects, we performed mixed ANCOVAs on the proportion of correctly localized peripheral targets with Valence (Positive vs Neutral vs Negative) and Distance (far vs close) as within-subject factors. When testing for moderation on the effect of positive mood induction on attentional breadth for valenced stimuli we entered Group (coded as 0 = neutral MIP, 1 = positive MIP) as between subjects variable. When testing for moderation on the direct effect of positive mood change on attentional breadth, the change in positive mood in response to the mood induction, across all participants (ΔPANASpositive) was entered as continuous independent variable (covariate). Additionally, BDI scores were entered as continuous independent variable (covariate). A custom model was then specified in order to be able to investigate Group × BDI × within-subject factor interactions or ΔPANASpositive × BDI × within-subject factor interactions (Group and ΔPANASpositive were entered in separate moderation analyses).

In case the mixed ANCOVA revealed a moderation effect, we performed a simple slope analysis to better understand the direction of the moderation effect of BDI on the relation between positive mood and attentional breadth for emotional information. We used Hayes and Matthes’ SPSS macro (Hayes and Matthes, 2009) for the simple slope analysis. This analysis estimates different conditional effects of the focal predictor (either Group or ΔPANASpositive) on the outcome variable (e.g., overall ANI or ANIpositive) at low (one SD below the mean), moderate (sample mean), and high (one SD above the mean) values of the moderator (BDI scores).

## RESULTS

### GROUP CHARACTERISTICS

Nineteen participants received the positive MIP and 19 participants received the neutral MIP. The means and standard deviations for the baseline variables can be seen in Table 1. Some variables (BDI) were not normally distributed and were therefore square root transformed before testing group differences with an independent t-test. As some variables remained not normally distributed after transformation (PANAS negative baseline, age) we performed non-parametric Mann–Whitney test for group differences on these variables. There were no significant differences between the groups in terms of gender, χ^2^ (1, *N* = 38) = 0.36, *p* = 0.547, age, and baseline measures, all p-values >0.30.

**Table 1:** **Group characteristics.**

	Neutral (*n* = 19)	Positive (*n* = 19)
	*M* (SD)	*M* (SD)
Age	20.79 (2.15)	20.84 (1.50)
Gender	17 females	18 females
PANAS positive baseline	33.42 (6.67)	34.37 (4.78)
PANAS negative baseline	13.84 (4.48)	13.47 (4.65)
BDI	6.63 (5.75)	6.26 (6.85)
State–Trait Anxiety Inventory	37.84 (14.48)	36.68 (9.59)
Ruminative Response Scale total	46.05 (9.47)	42.68 (12.67)
SUIS	41.11 (7.04)	40.79 (7.47)

### MOOD MANIPULATION CHECK

For the PANAS positive scale the mixed ANOVA with Group (Positive MIP vs Neutral MIP) as between subject factor and Time (before vs after the MIP) as within-subject factor revealed a significant main effect of Time, *F*(1,36) = 11.16, *p* = 0.002, η^2^ = 0.24, and a Group × Time interaction, *F*(1,36) = 5.11, *p* = 0.030, η^2^ = 0.12. A non-parametric Mann–Whitney test was performed to test for group differences in the change scores on the PANAS negative scale across the induction (i.e., the difference scores; data was not normally distributed). This test revealed no significant group difference, *U* = 164.50, *p* = 0.848, *r* = -0.03^2^.

The significant Group × Time interaction for the PANAS positive scale was driven by participants from the positive MIP group showing a significant increase in levels of positive affect from pre-MIP (*M* = 34.37) to post-MIP (*M* = 37.37), *t*(18) = 3.69, *p* = 0.002, while participants from the neutral MIP group did not show such an increase from pre-MIP (*M* = 33.42) to post-MIP (*M* = 34.00), *t*(18) = 0.83, *p* = 0.418. The positive MIP group tended to report significantly higher levels of positive affect afterward, *F*(1,36) = 3.39, *p* = 0.074, η^2^ = 0.09, which was significant when taking baseline mood levels into account, *F*(1,35) = 6.39, *p* = 0.016, η^2^ = 0.15.

### MOOD AND ATTENTION FOR EMOTIONAL INFORMATION

The 3 Valence (Positive vs Neutral vs Negative) × 2 Distance (far vs close) × 2 Group (Positive MIP vs Neutral MIP) mixed ANOVA yielded only a significant main effect of Distance, *F*(1,36) = 214.30, *p* < 0.001, η^2^ = 0.86; that is, across all participants the proportion of correctly localized targets was higher when the target appeared at close distance (M = 0.89, SD = 0.14) than when the target appeared at further distance (M = 0.45, SD = 0.20). All other F-values <1.50.

The ANCOVA with the ΔPANASpositive as a covariate yielded also only a significant main effect of Distance, *F*(1,36) = 157.56, *p* < 0.001, η^2^ = 0.81.

### MODERATION OF THE RELATION BETWEEN MOOD AND ATTENTION FOR EMOTIONAL INFORMATION

We further investigated possible moderation effects of depressive symptoms (as measured by the BDI) on the relation between positive mood and attentional breadth to test whether the presence of depressive symptoms influences the effects of positive mood on attentional broadening.

#### Moderation of depressive symptoms on relation between mip group and attentional breadth for emotional stimuli

The mixed ANCOVA on the proportion of correctly localized peripheral targets with Valence (Positive vs Neutral vs Negative) and Distance (far vs close) as within-subject factors, Group (0 = neutral MIP, 1 = positive MIP) as between subjects variable and BDI scores as continuous independent variable (covariate) revealed a near significant Distance × Group × BDI interaction, *F*(1,34) = 3.88, *p* = 0.057, η^2^ = 0.10. This indicates that the BDI tends to moderate the relation between Group and Attentional narrowing/broadening but regardless of the Valence of the central stimulus.

To better understand the direction of the moderation effect of BDI on the relation between Group and overall Attentional narrowing/broadening, we did a simple slope analysis for the overall ANI (i.e., regardless of whether the central stimulus was positive, neutral, or negative). Results from the simple slope analyses showed a positive relation between Group and the overall ANI only among high levels (above 1 SD) of BDI scores, *t* = 2.27, *p* = 0.030, *b* = 0.19. Such relation was absent (i.e., non-significant) for moderate levels (mean) of BDI scores, *t* = 1.22, *p* = 0.229, *b* = 0.07 and for low levels (below 1 SD) of BDI scores, *t* = -0.56, *p* = 0.580, *b* = -0.05. The simple slope analysis revealed no relation between positive mood and overall attentional narrowing/broadening at low and moderate levels of BDI scores. However, among high levels of BDI scores we found a relation between group and overall attentional breadth showing that participants in the positive MIP group showed more overall attentional narrowing as compared to the neutral MIP group.

#### Moderation of depressive symptoms on relation between change in positive mood and attentional breadth for emotional stimuli

The mixed ANCOVA on the proportion of correctly localized peripheral targets with Valence (Positive vs Neutral vs Negative) and Distance (far vs close) as within-subject factors, ΔPANASpositive and BDI scores as continuous independent variables (i.e., covariates) revealed a significant Distance × ΔPANASpositive × BDI interaction, *F*(1,34) = 8.98, *p* = 0.005, η^2^ = 0.21, but also a significant Valence × Distance × ΔPANASpositive × BDI interaction, *F*(2,33) = 4.65, *p* = 0.017, η^2^ = 0.22. This indicates that the moderation effect of the BDI on the relation between ΔPANASpositive and Attentional narrowing/broadening is different depending on the Valence of the central stimulus. There was no significant correlation between the BDI and ΔPANASpositive itself, *p* > 0.10.

To better understand the direction of this moderation effect and its interaction with Valence of the central stimulus, we performed simple slope analyses for the Attentional Narrowing Indices for positive, neutral, and negative central stimuli separately.

**FIGURE 2:**
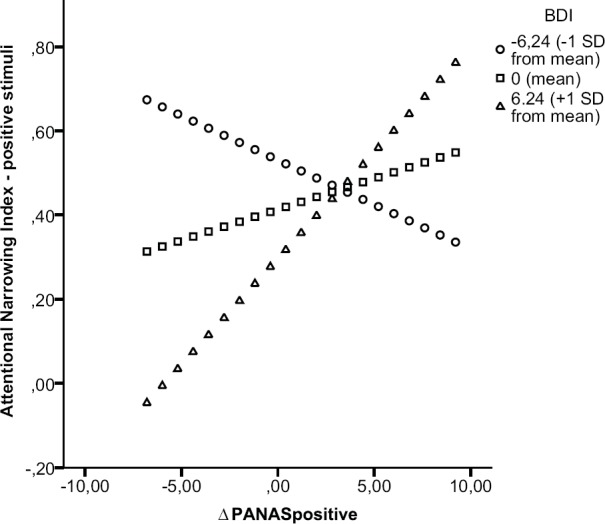
**Moderation effect of BDI on relation positive mood and attentional breadth for positive stimuli.** Simple slope analysis on the moderation effect of BDI on the relation between ΔPANASpositive and the Attentional Narrowing Index for positive central stimuli. Regression lines were plotted by substituting low, moderate, and high values of BDI scores using Hayes and Matthes’ SPSS macro (Hayes and Matthes, 2009).

***Attentional breadth for positive stimuli.*** Results of the moderation analysis on ANIpositive showed a significant moderation effect, the interaction term (ΔPANASpositive × BDI) was a significant predictor, *t* = 4.37, *p* < 0.001, and added an explained variance to the model of ΔR^2^ = 0.34. Results from the simple slope analyses (see Figure 2) showed a positive relation between the increase in positive mood and attentional narrowing for positive stimuli among high levels (above 1 SD) of BDI scores, *t* = 4.04, *p* < 0.001, b = 0.05, while this relation was only near significant, *t* = 1.83, *p* = 0.077, *b* = 0.02, among moderate levels (mean) of BDI scores. Among low levels (below 1 SD) of BDI scores a negative relation between the increase in positive mood and attentional narrowing for positive stimuli was shown, *t* = -2.04, *p* = 0.050, *b* = -0.02. Results from the simple slope analysis indicated that among high levels of depressive symptoms a bigger increase in positive mood was actually related to more attentional narrowing for positive stimuli, while among moderate levels of depressive symptoms only a near significant relation between positive mood and attentional breadth for positive stimuli was found. However, for low levels of depressive symptoms we found a relation between a bigger increase in positive mood and less attentional narrowing/more attentional broadening for positive stimuli.

**FIGURE 3:**
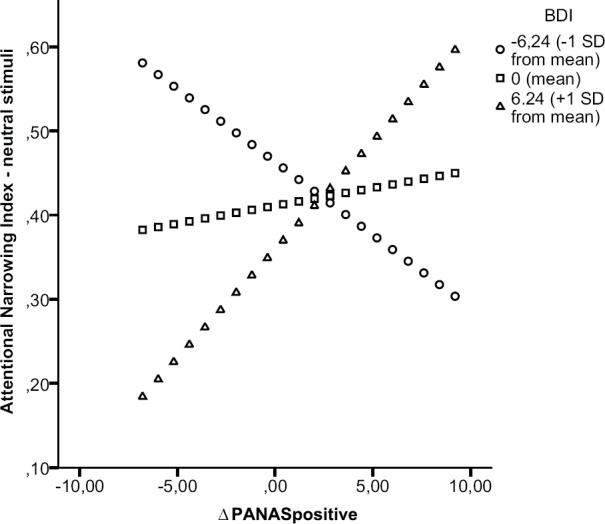
**Moderation effect of BDI on relation positive mood and attentional breadth for neutral stimuli.** Simple slope analysis on the moderation effect of BDI on the relation between ΔPANASpositive and the Attentional Narrowing Index for neutral central stimuli. Regression lines were plotted by substituting low, moderate, and high values of BDI scores using Hayes and Matthes’ SPSS macro (Hayes and Matthes, 2009).

***Attentional breadth for neutral stimuli.*** Results of the moderation analysis on ANIneutral showed a significant moderation effect, the interaction term was a significant predictor, *t* = 2.32, *p* = 0.027, and added an explained variance to the model of ΔR^2^ = 0.14. Results from the simple slope analyses (see Figure 3) showed only a marginally significant positive relation between the change in positive mood and attentional narrowing for neutral stimuli among high levels (above 1 SD) of BDI scores, *t* = 1.82, *p* = 0.078, *b* = 0.03. This relation was absent (i.e., not significant) among moderate levels (mean) of BDI scores, *t* = 0.46, *p* = 0.647, *b* = 0.004, and among low levels (below 1 SD) of BDI scores, *t* = -1.47, *p* = 0.151, *b* = -0.02. These results indicate that although the severity of depressive symptoms has a significant influence on the strength of the relation between positive mood and attentional narrowing for neutral stimuli, there is no significant relation between the change in positive mood and attentional breadth for neutral stimuli among moderate and low levels of BDI scores. Only among high levels of depressive symptoms results show a tendency for an increase in positive mood to be related to attentional narrowing for neutral stimuli.

**FIGURE 4:**
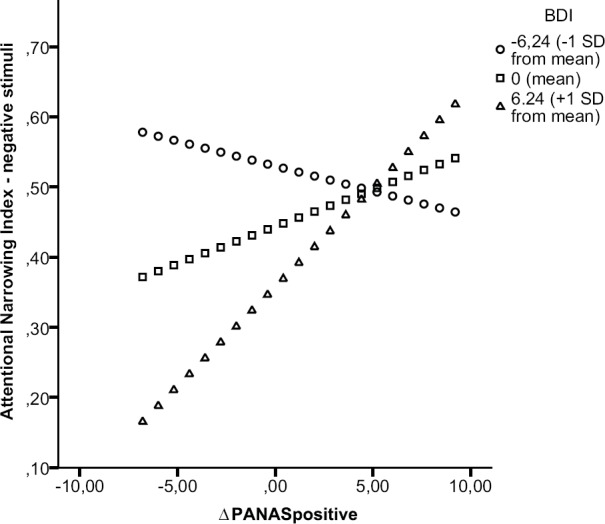
**Moderation effect of BDI on relation positive mood and attentional breadth for negative stimuli.** Simple slope analysis on the moderation effect of BDI on the relation between ΔPANASpositive and the Attentional Narrowing Index for negative central stimuli. Regression lines were plotted by substituting low, moderate, and high values of BDI scores using Hayes and Matthes’ SPSS macro (Hayes and Matthes, 2009).

***Attentional breadth for negative stimuli.*** Results of the moderation analysis on ANInegative showed only a trend for a moderation effect, *t* = 1.77, *p* = 0.086, with an added explained variance to the model of ΔR^2^ = 0.08. Simple slope analyses (see Figure 4) revealed only a marginally significant positive relation between the change in positive mood and attentional narrowing for negative stimuli among high levels (above 1 SD) of BDI scores, *t* = 1.86, *p* = 0.072, *b* = 0.03. Such a relation was absent (i.e., non-significant) among moderate levels (mean) of BDI scores, *t* = 1.08, *p* = 0.287, *b* = 0.01, and among low levels (below 1 SD) of BDI scores, *t* = -0.56, *p* = 0.578, *b* = -0.01. These results indicate that the level of depressive symptoms only tends to influence the relation between change in positive mood and attentional narrowing for negative stimuli. Only among high levels of BDI scores we found a trend for a bigger increase in positive mood to be related to more attentional narrowing for negative stimuli.

## DISCUSSION

The first aim of the current study was to investigate the relationship between positive mood and attentional breadth and to investigate whether this would interact with the emotional valence of the stimuli in the target of attention. Based on previous results from Wadlinger and Isaacowitz (2006) we expected that positive mood would be related to attentional broadening but specifically when the information in the target of attention is positive. However, our results did not confirm previous research as no direct relation was found between positive mood and attentional breadth, neither did this depend on the emotional valence of the presented information. These results are not in line with earlier findings (e.g., Fredrickson and Branigan, 2005) which confirmed the proposed broadening effect of the broaden-and-build theory (Fredrickson, 1998, 2001). Neither are our results fully consistent with earlier findings suggesting that the broadening effects of positive emotions are dependent on the emotional valence of the presented information (Wadlinger and Isaacowitz, 2006).

We further investigated whether the presence and severity of depressive symptoms could have an influence on the strength of the relationship between positive mood and attentional broadening. The broaden-and-build theory (Fredrickson, 1998, 2001) postulates that positive emotions are related to broadened cognition and it has been suggested that this broadening function plays an important role in explaining the relation between positive emotions and resilience against stress and possibly mood disorders. This would suggest that resilient people can benefit more from positive emotions at the cognitive level or more specifically, show the proposed broadening effects. On the other hand, vulnerability for mood disorders such as the presence of depressive symptoms (Fergusson et al., 2005), could have opposite moderating effects. Moreover, depressive symptoms have previously been associated with a more narrowed attentional scope (Basso et al., 1996; De Fockert and Cooper, 2014). It could be that the presence and severity of depressive symptoms influence the relation between positive mood and attentional breadth by hampering the cognitive effects of positive mood. The results from the moderation analyses with the BDI as a self-report measure of the presence and severity of depressive symptoms (in the last 2 weeks), showed that the BDI indeed moderated the relation between positive mood and attentional breadth. Results showed that the severity of depressive symptoms reversed the proposed direction of the relation. Among high levels of BDI scores—one standard deviation above the mean which was around the cut-off score for mild depressive symptoms (i.e., ≥14; Beck et al., 1996)—an increase in positive mood was related to more attentional narrowing in general, that is, regardless of the emotional valence of the presented stimuli. When analyzing the results of positive mood on a group level, that is, comparing participants from the positive and neutral MIP condition, we did not find evidence for the proposed relation between positive mood and attentional broadening among low and moderate levels of BDI scores.

However, when investigating the effects of positive mood in a more direct manner, that is, investigating the effects of the change in positive mood across the induction in the entire sample (an interindividual differences approach), we found that the BDI moderates the relationship between positive mood and attentional breadth, depending on the emotional valence of the presented stimuli. Among high levels of the BDI we found that an increase in positive mood was related to attentional narrowing when positive stimuli were presented, and this relation was only marginally significant when neutral and negative information were presented. Among moderate levels of the BDI we did not find a relation between positive mood and attentional broadening/narrowing, regardless of the emotional valence of the presented stimuli. Interestingly, however, when positive stimuli were presented we found that among low levels of the BDI, reflecting minimal depressive symptoms (i.e., ≤13; Beck et al., 1996), a bigger increase in positive mood was related to more attentional broadening. This is in line with previous research showing the broadening effect of positive emotions solely when positive information was presented (Wadlinger and Isaacowitz, 2006), and suggests that the presentation of negative information in attention might hamper the attentional broadening effects of a positive mood. However, results from our study indicate that individual characteristics, such as depressive symptoms, should be considered as well. These results may suggest that, at low levels of the BDI, a positive mood state is not necessarily related to general attentional broadening but flexible adaptation of the attentional scope on a trial-by-trial bias depending on the valence of the presented emotional information.

The current results on the moderating effect of depressive symptoms showed that among high levels of the BDI, around the cut-off for mild depressive symptoms (Beck et al., 1996), an increase in positive mood was related to attentional narrowing when positive stimuli were presented (although near significant for neutral and negative stimuli). This contrasts the broadening hypothesis, but could be seen in light of recent studies suggesting a flexible link between affect and attentional scope (Huntsinger et al., 2010; Huntsinger, 2012, 2013). This theoretical view proposes that affect, instead of having fixed effects on cognition, provides information about the attentional orientation that is most accessible or dominant at that moment (Clore and Huntsinger, 2007, 2009; Huntsinger, 2013). That is, positive affect is believed to act as a “go signal” for the use of the currently available or dominant mode of processing, while negative affect acts as a “stop signal” inhibiting this process (Huntsinger, 2012, 2013). Evidence for this view was found in two recent studies showing that positive mood encouraged either a global or local attentional focus, depending on which focus was made momentarily dominant by a priming procedure (Huntsinger et al., 2010; Huntsinger, 2012). As depressive symptoms have previously been related to a more narrow attentional scope (Basso et al., 1996; De Fockert and Cooper, 2014), it is possible that the positive mood induction in the current study encouraged the more dominant narrow attentional focus among higher BDI scores. However, this is just a tentative explanation and further research would be necessary to investigate the interactive relations between individual characteristics, momentary mood and cognition.

In the current study, we investigated how the presence of depressive symptoms would influence the cognitive effects of positive emotions, but a possible limitation of this study is the fact that we tested an unselected student sample which limited the number of participants with higher BDI scores. However, in the simple slope analyses, results are calculated at a high level of BDI scores—one standard deviation above the sample mean—which in the current sample was around the cut-off score for mild depressive symptoms (Beck et al., 1996) from where individuals are usually considered to be dysphoric. Moreover, previous studies (e.g., Fergusson et al., 2005) have shown that subthreshold levels of depression are a risk factor for the development of later depression. Thus, given that our results show a moderation effect of the BDI on the relation between positive mood and attentional breadth, it seems that interindividual variability in mostly non-clinical BDI scores already influences the strength and direction of the relation between positive emotions and cognition.

In summary, we did not find evidence that positive mood was directly related to attentional broadening, neither that this was influenced by the emotional valence of the information presented. However, moderation analyses revealed a moderation effect by the presence of depressive symptoms. Among low levels of BDI scores, reflecting minimal depressive symptoms, we did observe a relation between an increase in positive mood and attentional broadening specifically when positive stimuli were presented in the target of attention. Among moderate levels of BDI scores such a relation was absent regardless of the valence of the presented stimuli, while among high levels of BDI scores, around the cut-off for mild depressive symptoms, a reversed relation was observed with an increase in positive mood being related to attentional narrowing. This shows that individual characteristics such as the presence and severity of depressive symptoms, which indicate a vulnerability for mood disorders, influence the strength and even direction of the relation between positive mood and attentional breadth. Current results suggest that the cognitive broadening effects of positive emotions are not so straightforward and could be influenced by several factors like characteristics of the information presented, but also by individual characteristics such as the presence of depressive symptoms which have a hampering effect.

### Conflict of Interest Statement

The authors declare that the research was conducted in the absence of any commercial or financial relationships that could be construed as a potential conflict of interest.

## Footnotes

1 We also administered the trait version of the State–Trait Anxiety Inventory (Spielberger et al., 1983; Van der Ploeg et al., 2000) and the Ruminative Response Scale (Nolen-Hoeksema and Morrow, 1991; Raes et al., 2003), but these were not used to test the hypotheses of the current study.

2 A parametric mixed ANOVA with Time (pre- and post-MIP) as within-subject factor and Group as between-subject factor on the PANAS negative scale did not yield a significant Time × Group interaction either.
